# *CDHR1* mutations in retinal dystrophies

**DOI:** 10.1038/s41598-017-07117-8

**Published:** 2017-08-01

**Authors:** Katarina Stingl, Anja K. Mayer, Pablo Llavona, Lejla Mulahasanovic, Günther Rudolph, Samuel G. Jacobson, Eberhart Zrenner, Susanne Kohl, Bernd Wissinger, Nicole Weisschuh

**Affiliations:** 1 Institute for Ophthalmic Research, Centre for Ophthalmology, University of Tuebingen, Tuebingen, Germany; 2 CeGaT GmbH and Praxis fuer Humangenetik Tuebingen, Tuebingen, Germany; 3 University Eye Hospital, Ludwig Maximilians University, Munich, Germany; 4 Scheie Eye Institute, Department of Ophthalmology, Perelman School of Medicine, University of Pennsylvania, Philadelphia, PA USA; 5 Werner Reichardt Centre for Integrative Neuroscience (CIN), University of Tuebingen, Tuebingen, Germany

## Abstract

We report ophthalmic and genetic findings in patients with autosomal recessive retinitis pigmentosa (RP), cone-rod dystrophy (CRD) or cone dystrophy (CD) harboring potential pathogenic variants in the *CDHR1* gene. Detailed ophthalmic examination was performed in seven sporadic and six familial subjects. Mutation screening was done using a customized next generation sequencing panel targeting 105 genes implicated in inherited retinal disorders. In one family, homozygosity mapping with subsequent candidate gene analysis was performed. Stringent filtering for rare and potentially disease causing variants following a model of autosomal recessive inheritance led to the identification of eleven different *CDHR1* variants in nine index cases. All variants were novel at the time of their identification. *In silico* analyses confirmed their pathogenic potential. Minigene assays were performed for two non-canonical splice site variants and revealed missplicing for the mutant alleles. Mutations in *CDHR1* are a rare cause of retinal dystrophy. Our study further expands the mutational spectrum of this gene and the associated clinical presentation.

## Introduction

Significant progress has been made over the past two decades in defining the molecular pathogenesis of inherited retinal dystrophies (IRDs), a group of diseases characterized by gradual loss of photoreceptor cells and resulting in compromised vision often leading to patients being registered as blind. IRDs can be broadly characterized on the basis of the sequential order and relative severity of the dysfunction or loss of either rods or cones, and are therefore subdivided in cone dystrophies (CD), cone–rod dystrophies (CRDs) and rod-cone dystrophies (also known as retinitis pigmentosa; RP)^1^. Patterns of inheritance include autosomal recessive, autosomal dominant and X-linked forms. Moreover rare mitochondrial and digenic forms of retinal dystrophies have also been reported. Initial symptoms of cone dysfunction include early and progressive loss of central visual acuity, photophobia and defects in color vision^2^. If the primary insult affects the rod system, the first noticeable symptom is usually night blindness; visual field defects start in the mid-periphery and then proceed centrally and peripherally, while macular vision remains relatively preserved until the cones undergo secondary degeneration^3^. A fact that often hampers clinical and genetic diagnosis is that IRDs display pronounced locus heterogeneity, allelic heterogeneity as well as clinical heterogeneity. To date, mutations in more than 125 genes have been implicated in non-syndromic forms of IRDs (https://sph.uth.edu/retnet/). The function of these IRD genes is rather diverse, including those that encode proteins of the phototransduction cascade, structural proteins, ciliary proteins, transcription and splicing factors and others. One of the genes associated with IRDs that plays a key role in the maintenance of photoreceptor structure and integrity is the cadherin-related family member 1 gene (*CDHR1*, OMIM 609502). The encoded protein is a protocadherin and belongs to the group of the cadherin superfamily of homophilic cell-adhesion proteins. Protocadherins are predominantly expressed in the nervous system, and constitute the largest subgroup in the cadherin superfamily^4^. Beside *CDHR1*, three other cadherin family members have been found to be implicated in retinal dystrophies^5–7^. CDHR1 is composed of six cadherin repeats, one transmembrane, and one intracellular domain. The gene product is found in several tissues, but highest abundance is in the retina^8, 9^. Within the retina, CDHR1 predominantly localizes at the junction between the inner- and outer segments of rod and cone photoreceptors^10, 11^. A *Cdhr1* knockout mouse shows disorganization of photoreceptor outer segments and a progressive loss of photoreceptor cells^11^. Apart from the variants described in this study, 20 mutations in *CDHR1* that explain the disease phenotype in the respective patients have been associated with retinal dystrophies to date^12–25^. Most variants presumably lead to functional null alleles. The phenotype of the patients shows broad variability with respect to onset and primary insult; diagnoses range from cone dystrophy to retinitis pigmentosa.

In this study, we describe novel genetic findings in an international cohort of 13 probands from nine unrelated families diagnosed either with CRD, CD or RP. Patients were investigated in one case by autozygosity mapping with subsequent candidate gene analysis and in the other cases by using a next generation sequencing approach. Patients also underwent detailed clinical evaluation by multimodal ophthalmological diagnostics (retinal imaging, functional tests and electroretinography techniques).

## Results

### Genotyping

**Figure 1:**
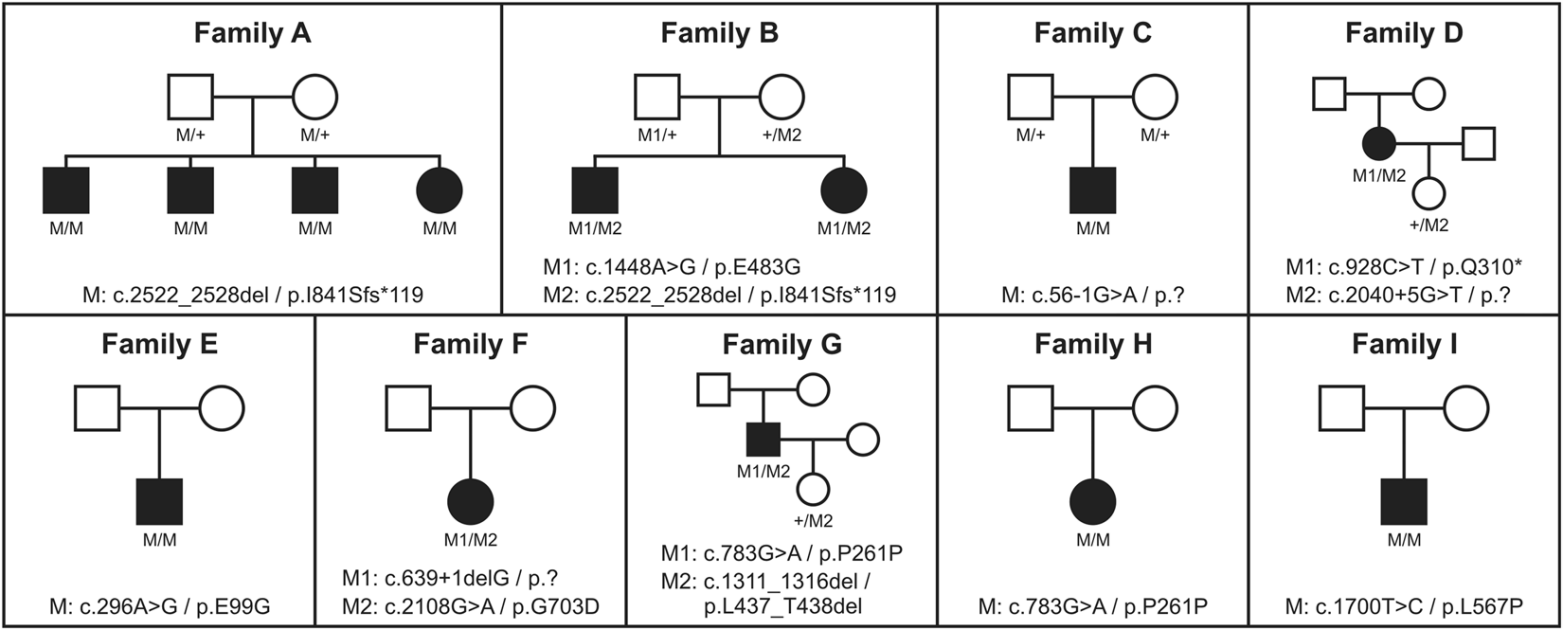
Pedigrees of all families. Disease-causing mutation(s) are given below each pedigree and the genotypes of all available family members.

Homozygosity mapping of three affected siblings from family A revealed a 3.5-Mb shared homozygous interval (Chr10.hg19:g.85,612,092–89,472,813). The interval comprises 45 HUGO Gene Nomenclature Committee (HGNC)-approved protein-coding genes, among them *CDHR1*. Subsequent sequence analysis revealed a homozygous 7 bp-deletion in *CDHR1*, c.2522_2528del/p.I841Sfs*119, that segregated in the family (Fig. 1). Patients from families B-I were analyzed by means of a targeted capture panel of retinal disease genes. In all eight cases, rare and potentially disease-causing variants compatible with a model of autosomal recessive inheritance were only observed in the *CDHR1* gene, but not in other genes associated with retinal dystrophies. In four cases, the disease-causing variant was in apparent homozygous state. Segregation analysis confirmed heterozygosity in both parents in family C (Fig. 1), whereas parental samples were not available for the other three families (E, H and I). The probands from the remaining four families (B, D, F and G) showed two heterozygous variants in the *CDHR1* gene each and compound heterozygosity could be demonstrated for families B, D and G where additional family members were available (Fig. 1). Compound heterozygosity could not be demonstrated for patient F and we have to point out that her phenotype might not be *CDHR1*-related, although no putative disease-causing variants in other retinal disease genes were identified in the panel sequencing approach. All variants were novel at the time of identification but those variants identified in families B, D, G and H have already been included in the publication of cohort screening studies^26, 27^. The variants we identified in this study comprise four missense, one nonsense, one frameshift deletion, and one in frame deletion. Four additional variants were found at or close to the canonical splice sites. None of the variants was observed in the ExAC database with the exception of the canonical splice site variant c.56–1 G > A (MAF = 0.00092%) and the exonic splice variant c.783 G > A/p.P261P (MAF = 0.34%). The frameshift deletion variant c.2522_2528del/p.I841Sfs*119 that was seen in homozygous state in family A, was also seen in (compound) heterozygous state in family B. The splice site variant c.783 G > A/p.P261P that was seen in homozygous state in family H was also seen in (compound) heterozygous state in family G. The remaining variants were unique.

To evaluate the pathogenicity of the missense and splice site variants, we performed *in silico* analyses using different prediction programs (see Table 1). The four missense variants p.E99G, p.E483G, p.L567P and p.G703D, affect amino acid residues that are highly conserved among vertebrate species. Accordingly, all four missense variants are predicted to have a damaging effect on protein function by at least three prediction programs. Two variants affect canonical splice sites (c.56–1 G > A and c.639 + 1delG) and are therefore likely to have a major effect on splicing. *In silico* assessment using five algorithms embedded in the Alamut software indeed predicted the virtual abolishment of the respective splice sites.

**Table 1:** Pathogenicity predictions for missense and splice site mutations based on *in silico* analyses.

Amino acid change predictions							
DNA change	Protein change	ExAC (MAF)	SIFT [0–1]	Provean*	Polyphen-2 [0–1]	Mutation Taster [0–1]	PhyloP [−14.1;6.4]
c.296 A > G	p.E99G	—	Deleterious (0.0)	Deleterious (−5.6)	Probably damaging (1.0)	Disease causing (1.0)	4.08
c.1311_1316del	p.L437_T438del	—	N/A	Deleterious (−11.5)	N/A	Disease causing (0.87)	N/A
c.1448 A > G	p.E483G	—	Deleterious (0.0)	Deleterious (−6.1)	Probably damaging (1.0)	Disease causing (1.0)	4.56
c.1700T > C	p.L567P	—	Deleterious (0.0)	Deleterious (−4.0)	Probably damaging (1.0)	Disease causing (1.0)	1.58
c.2108 G > A	p.G703D	—	Deleterious (0.01)	Neutral (−2.4)	Probably damaging (0.99)	Disease causing (1.0)	4.08
**Splicing predictions**							
**DNA change**	**Predicted effect**	**ExAC (MAF)**	**SSF [0–100]**	**MaxEnt [0–12]**	**NNSPLICE [0–1]**	**GeneSplicer [0–15]**	**HSF [0–100]**
c.56-1 G > A	Broken acceptor site	0.00092%	92.71 → 0	10.34 → 0	0.98 → 0	12.64 → 0	93.45 → 0
c.639 + 1delG	Broken donor site	—	81.67 → 0	8.92 → 0	0.95 → 0	10.23 → 0	84.70 → 0
c.783 G > A	Broken donor site	0.34%	76.64 → 0	7.85 → 3.07 (−60.9%)	N/A	5.87 → 1.89 (−67.8%)	85.75 → 75.17 (−12.3%)
c.2040 + 5 G > T	Broken donor site	—	92.58 → 80.19 (−13.4%)	10.03 → 5.41 (−46.1%)	1.00 → 0.81 (−18.3%)	10.35 → 6.32 (−39.0%)	97.08 → 84.76 (−12.7%)

ExAC, Exome Aggregation Consortium; MAF, minor allele frequency. *A score threshold of −2.5 was used. N/A, not applicable.

### Functional analyses

In one patient (family G), we found compound heterozygosity for a 6-bp deletion in exon 12 (c.1311_1316delAACCTT), predicting the loss of a leucine and a threonine residue (p.L437_T438del), and a transition of the last nucleotide of exon 8 (c.783 G > A). The two lost amino acid residues are located in the fourth cadherin repeat and are fully conserved in vertebrates. The deletion is predicted to be disease-causing by all variant assessment algorithms (Table 1). The second variant in this patient changes the last nucleotide of exon 8 and thus may interfere with normal splicing. The last exonic nucleotide at an exon- intron boundary is usually guanine, and mutations at this position have been shown to result in splicing defects^28^. Indeed, computer-assisted analysis predicted a significant weakening of the donor splice-site consensus sequence of exon 8, although the predicted effect on splicing is much smaller when compared to the canonical splice site variants (Table 1). The same holds true for the variant c.2040 + 5 G > T found in patient D since the +5 position is not invariable. Hence, we decided to investigate the potential effect on splicing for the two non-canonical splice site variants c.783 G > A and c.2040 + 5 G > T in more detail. Owing to the lack of *CDHR1* expression in blood cells, we made use of a heterologous splicing assay in HEK 293 T cells to test mutant and wild-type *CDHR1* minigene constructs in direct comparison. A minigene construct comprising parts of *CDHR1* from intron 6 to intron 9 was generated to contain either the c.783 A mutant or the c.783 G control, respectively. Accordingly, a minigene construct comprising a fragment from intron 14 to intron 16 of *CDHR1* was generated to contain either the c.2040 + 5 T mutant or the c.2040 + 5 G control, respectively. Sanger sequencing revealed no further sequence difference between the mutant and the control constructs.

**Figure 2:**
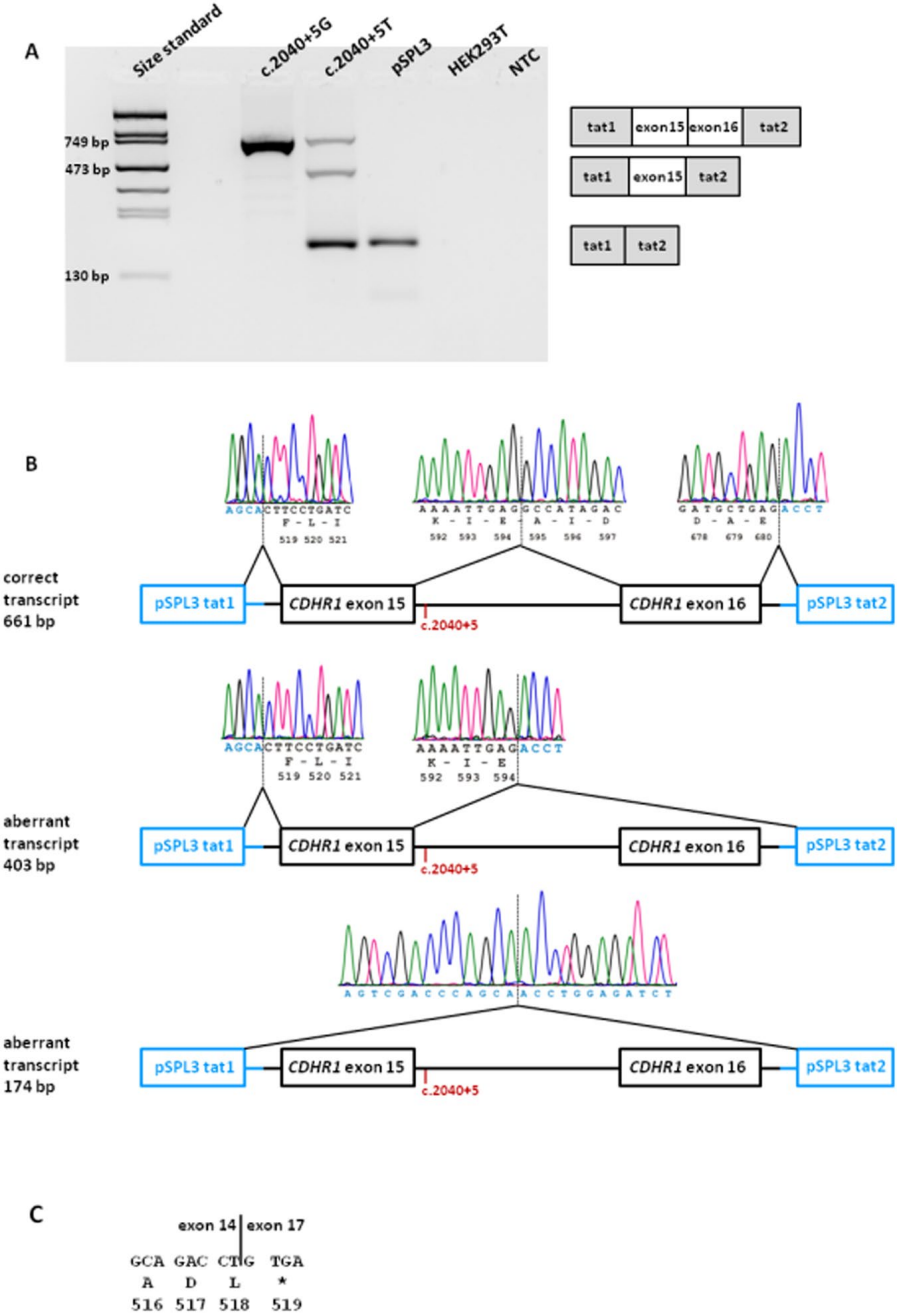
*In vitro* splicing assay for the c.2040 + 5 G > T variant. (**A**) RT–PCR revealed three products in HEK 293 T cells transfected with the mutant construct and one product for the wild-type construct. Transfection with empty pSPL3 vector and untransfected cells served as controls. NTC, non template control. Schemes of the amplified products are presented on the right of the agarose gel. Grey boxes represent pSPL3 exons and white boxes *CDHR1* exons. (**B**) Sequence analysis showing that one aberrant RT-PCR product from the mutant minigene construct results from skipping of exon 16 and the other aberrant RT-PCR product results from skipping of both exon 15 and 16. The position of the c.2040 + 5 G > T variant is depicted in red. (**C**) Consequence of the skipping of exon 15 and 16 on protein level. Mutant CDHR1 transcript lacking exons 15 and 16 will contain a premature stop codon (protein translation is shown below DNA sequence in one letter amino-acid code).

Upon transfection of the minigene constructs in HEK 293 T cells, RT–PCR of c.2040 + 5 G RNA using pSPL3 backbone primers resulted in a single product of the expected size (Fig. 2A), and subsequent sequencing confirmed correct splicing of exons 15 and 16 (Fig. 2B). In contrast, RT–PCR of c.2040 + 5 T RNA revealed three different products of different proportions (Fig. 2A). Subsequent sequencing showed that one of these RT–PCR products derived from the transfection with the mutant c.2040 + 5 T -allele lacked exon 16. The aberrant transcript would lead, if translated, to a shortened protein that lacks the last cadherin repeat. The smallest and most prominent transcript lacks both exon 15 and 16 and would lead, if translated, to a premature stop codon (Fig. 2C). Sanger sequencing also revealed a minor portion of correctly spliced transcript.

**Figure 3:**
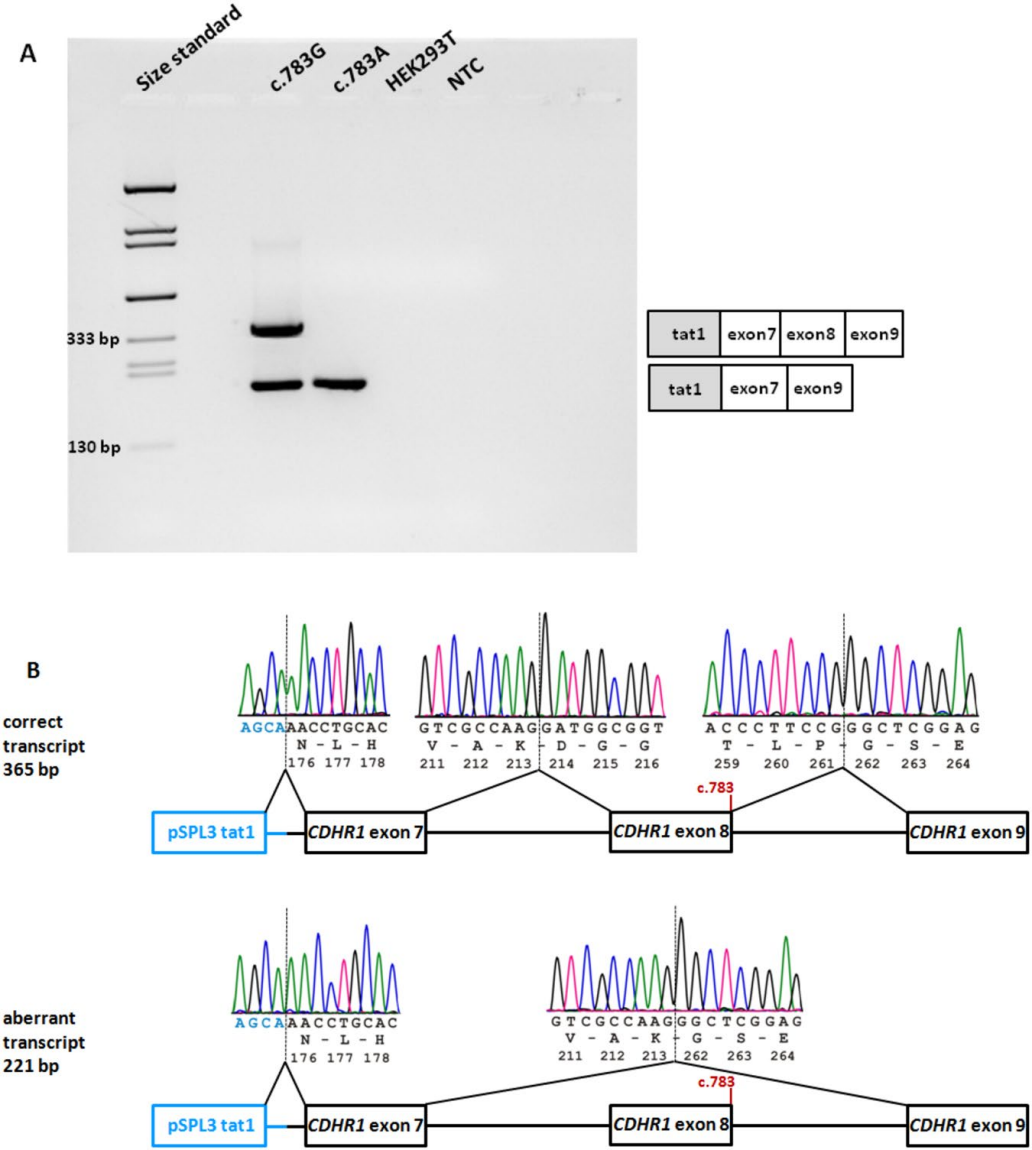
*In vitro* splicing assay for the c.783 G > A variant. (**A**) RT-PCR revealed two products in HEK 293 T cells transfected with the wild-type construct and a single RT-PCR product for the mutant construct. Untransfected cells served as control. NTC, non-template control. Schemes of the amplified products are presented on the right of the agarose gel. Grey boxes represent pSPL3 exons and white boxes *CDHR1* exons. (**B**) Sequence analysis showing that the aberrant product results from skipping of exon 8. The position of the c.783 variant is depicted in red. The junction of *CDHR1* exon 9 and pSPL3 tat2 could not be shown since a reverse PCR primer was used that binds within exon 9.

Upon transfection of the minigene constructs in HEK 293 T cells, RT-PCR of c.783 G RNA resulted in two products (Fig. 3A). Subsequent sequencing showed that the larger RT-PCR product corresponds to the correctly spliced transcript whereas the smaller RT-PCR product lacked exon 8 (Fig. 3B). RT-PCR of c.783 A RNA resulted in a single product which was sequenced and showed skipping of exon 8.

### Patient phenotypes

In total, 13 patients (eight males, five females, mean age at presentation 42 ± 13.2 years) were phenotyped. If patients were known and followed up for a longer time, only the last, most current follow-up visit is presented here.

Based on the clinical examinations with an emphasis on the full field ERG results, six patients were diagnosed with cone-rod dystrophy (all members of families A and B), three with cone dystrophy (families D, E and H) and four with retinitis pigmentosa (families C, F, G and I). Disease-onset was 25.3 ± 16.5 years; in addition, incidental findings in two patients led to a diagnosis at 20 and 50 years, respectively.

**Figure 4:**
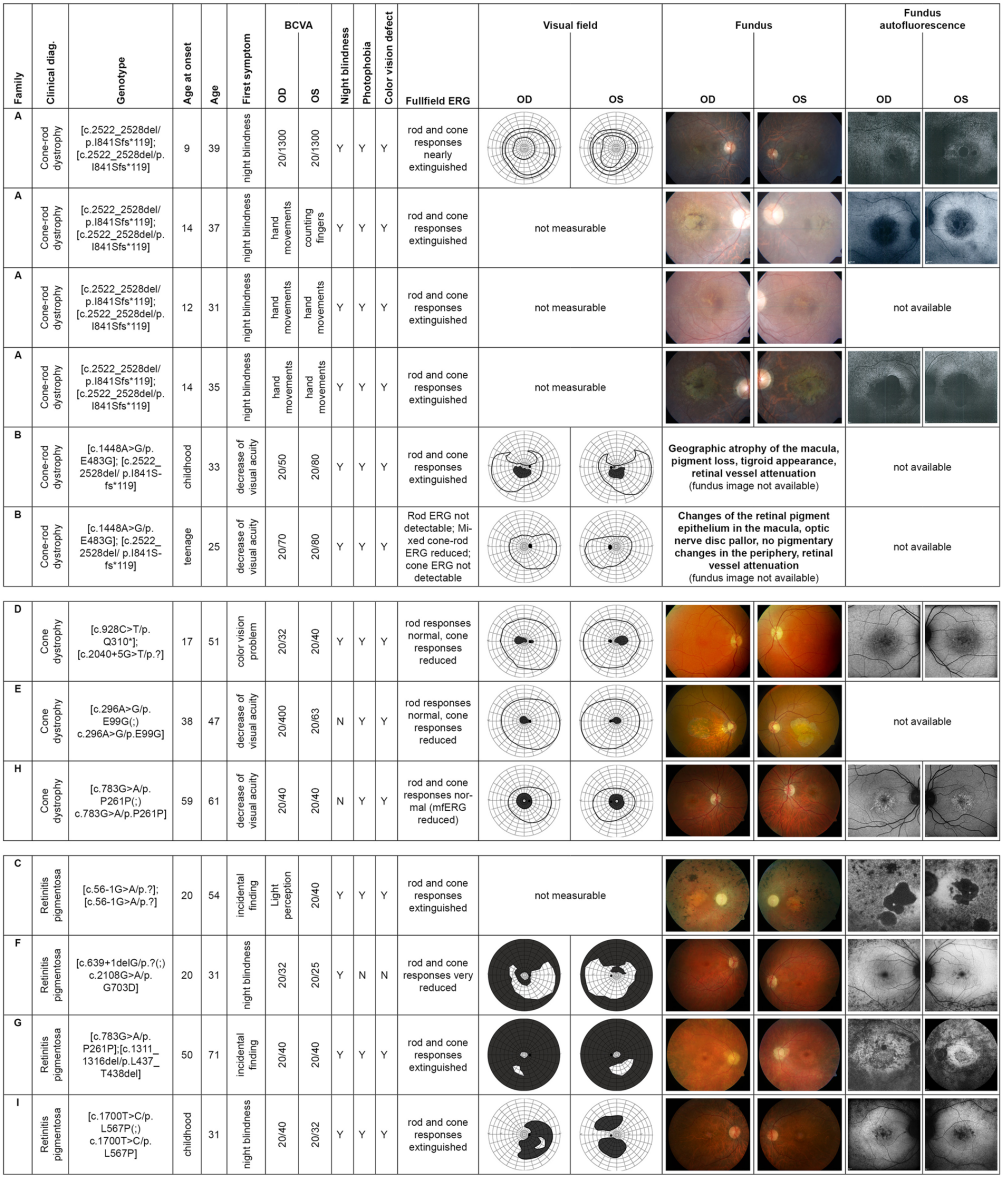
Summary of clinical findings. The composite image shows the most important available clinical findings. Each row represents a single patient with the following information in each column: Family (affiliation to the family according to Fig. 1); clinical diagnosis (CRD, CD or RP); genotype; age of onset (as reported subjectively by the patient); age (at examination); first symptom (first manifestation of the disease as reported by the patient); BCVA (best corrected visual acuity); night blindness, photophobia, color vision defect (“Y” for present, “N” for absent); fullfield ERG; visual field (if available); fundus image (if available); fundus autofluorescence image (if available).

An overview of the clinical diagnoses, age at onset, age at presentation, BCVA, typical symptoms (night blindness, photophobia, color vision defects), full field ERG findings, visual fields, fundus images and fundus autofluorescence images (FAF), and the genotypes of the patients are given in Fig. 4. Some patients were followed up for several years and disease progression was observed in all of them.

#### Cone dystrophies (CD) and cone-rod dystrophies (CRD)

The mean age of disease onset in CD/CRD was 23.2 ± 17 years with first subjective symptoms being decreased visual acuity or color vision problems in all but one family. Members of family A had night blindness as the first reported symptom at disease onset, and this was followed by central vision problems a few years later. With age at presentation between 25 and 59 years the BCVA ranged from hand movements detection to 20/32. All CD/CRD patients suffered from color vision disturbances, photophobia and night blindness with the exception of patient D (only minimal subjective night blindness) and patient E and H (no night blindness; confirmed by normal FST results with −49 dB and −47 dB, respectively, for white light). Accordingly, the rod ERGs of the three patients D, E and H showed normal responses, with reduced cone ERGs in patients D and E and only reduced multifocal ERG in patient H, whereas both cone and rod responses in the patients with cone-rod dystrophies were extinguished or markedly reduced. Visual fields were either not measurable or showed central scotomas in CRD/CD patients. Patient H had some limitation of the peripheral field and a pericentral scotoma. Fundus examination revealed a macular atrophy of different extent ranging from changes visible only in OCT and FAF (patient D and H) to geographic atrophy (two members of family A, one member of family B, patient E). A disturbed layer of the cone outer segments and retinal pigment epithelium was visible in all patients via OCT and/or FAF, but patients D and H presented only with subtle changes resembling an occult macular dystrophy. In the anterior segments mild cortical or subcortical opacities of the lens were present in families A, B, and E, otherwise the optical media were clear. One member of family A and both members of family B showed nystagmus. The patient from family D reported a latent strabismus. The right eye of patient E was amblyopic. Both patients from family B had hypercholesterinemia despite a relatively young age. Patient E suffered from diabetes mellitus and coronary heart disease; patient H had hypertension. No other medical history was reported among the patients with CD/CRD.

#### Retinitis pigmentosa (RP)

The mean age of disease onset in RP was 30 ± 14.1 years with the symptoms of night blindness in patients F and I, and incidental findings in patients C (at 20 years) and G (at 50 years). With age at presentation between 31 and 71 years the BCVA ranged from light perception to 20/25. All patients reported night blindness at presentation. Color vision disturbances and photophobia were reported in all patients except patient F (the youngest patient in the cohort), who suffered from neither of them. The full field ERGs of the patients were extinguished, except for patient F, whose fullfield ERG at age 31 was still measurable, but markedly reduced in both cone and rod responses. Visual fields were measurable only in three patients (F, G and I), showing ring scotomas of various degrees. Fundus examination revealed typical bone spicule-like pigment in the mid-periphery and periphery in all patients. In patient F white dots and atrophy were also present to a limited extent. Atrophic macular changes were visible on funduscopy of patients, ranging from geographic atrophy (patient C) or bulls-eye-like changes (patient G) to more subtle changes such as macular pucker (patient I) and an absent foveolar reflex (patient F). In FAF however, changes of the macular/foveal region were visible in all patients. OCT examination showed reduced photoreceptor outer segments up to the fovea in patients F, G and I (the less progressed cases), and rather diffuse outer retinal atrophy in patient C. All patients developed subcapsular cataract or had intraocular lenses already at presentation. In the youngest patient (F) the lens changes were only mild. The anterior segments were clear. Patient I was myopic (cca −7 dpt) prior to the intraocular lens surgery. Systemic hypertension was reported in two patients (C and G). Patient G also suffered from sleep apnea.

## Discussion

### Mutations in *CDHR1* lead to variable phenotypes

The phenotypes from our 13 patients demonstrate that mutations in the *CDHR1* gene can lead both to retinitis pigmentosa (RP) and cone dystrophies (CD) or cone-rod dystrophies (CRD). The clinical picture of these retinal degenerations is heterogeneous, as are the accompanying genotypes. However, a common aspect of all the phenotypes observed in our cohort seems to be macular involvement, not only in the CD/CRD patients, but also in the RP cohort. The clinical findings of our four RP patients show that the rod-cone retinal degeneration caused by mutations in the *CDHR1* gene is of the RP type with early macular involvement. The macular region was altered in all patients examined regardless of the disease duration, as seen in FAF, even in the youngest patient (F), who has a well-preserved visual acuity and measurable, albeit reduced, cone ERG. In the two oldest patients of the cohort the macular pigment changes as seen in the FAF were most pronounced (Fig. 4). In addition, color vision disturbances and photophobia, both signs of cone involvement, were present in all patients but the youngest one (F) who might be an example of a rather early stage of RP caused by mutations in the *CDHR1* gene, having a relatively well-preserved central vision and measurable ERG at the age of 31. Taken together, an early maculopathy might be a symptom to be expected in all patients with *CDHR1*-related retinopathy regardless of age.

Our cohort comprises two families with two or more affected siblings (families A and B). Of note, their clinical findings reveal a similar pattern, although the intrafamiliar findings are not identical. For example, two patients from family A have a geographic macular atrophy, whereas the other two siblings only show an absent reflex in the macula on funduscopy. These differences do not correlate with age. Similarly, only one sibling from family B has a clear geographic atrophy.

We found three patients with no objective affection of the rod system in our cohort (patients D, E and H). Interestingly, these are the three oldest subjects in the CD/CRD group. The diagnosis of cone dystrophy was confirmed by a normal rod ERG and no history of night blindness in two patients (E and H) and only a very slight subjective night blindness in patient D, which may still be in the normal range of dark adapted vision, although dark adaptation threshold testing was not performed in this patient. Patient H showed a constriction of the peripheral visual field in the light-adapted cone related kinetic visual field measurement, a process that assesses cone function only. Apparently the patient belongs to a group in which only cones are affected, with degeneration starting in the retinal periphery and pericentral areas, leaving central cones, which are the majority of cones dominating the ERG, intact for a longer time.

### Genotype-phenotype correlation

A clear association between the genotype and the clinical presentation is difficult to establish in our cohort since a number of patients carry two different mutations. In addition, the potential effect on protein function of most of the eleven different variants we identified in this study is difficult to predict. While the nonsense variant (c.928 C > T/p.Q310*) and the frameshift deletion variant (c.2522_2528del/p.I841Sfs*119) can be considered to represent loss of function alleles, the two variants that affect canonical splice sites (c.56-1 G > A and c.639 + 1delG) might confer residual protein function since the skipping of the respective exons would not alter the reading frame. However, without cDNA analysis it is not possible to predict the consequence to RNA processing which could just as well be intron inclusion leading to a change of the reading frame. The consequence of the four missense variants (c.296 A > G/p.E99G, c.1448 A > G/p.E483G, c.1700T > C/p.L567P and c.2108 G > A/p.G703D) and the in frame deletion (c.1311_1316del/p.L437_T438del) on protein function is also difficult to predict. All of these affected amino acid residues reside within the six Cadherin repeat-like domains with the exception of the glycine residue at amino acid position 703 which is the start of the transmembrane domain. However, due to the lack of functional studies a pathogenic effect can only be deduced from the rarity (none of the missense variants is reported in ExAC), *in silico* predictions and the high level of evolutionary conservation (Table 1). We have applied the variant classification terminology from the American College of Medical Genetics and Genomics (ACMGG) and the Association for Molecular Pathology (AMP) in order to give a better orientation with respect to the possible pathogenicity of all variants we have identified (Table 2)^29^.

**Table 2:** Classification of all *CDHR1* variants identified in this study according to ACMG standards.

DNA change	Protein change	Classification
c.56-1 G > A	p.?	Pathogenic
c.296 A > G	p.E99G	Uncertain significance
c.639 + 1delG	p.?	Pathogenic
c.783 G > A	p.P261P/p.?	Uncertain significance
c.928 C > T	p.Q310*	Pathogenic
c.1311_1316del	p.L437_T438del	Likely pathogenic
c.1448 A > G	p.E483G	Likely pathogenic
c.1700T > C	p.L567P	Uncertain significance
c.2040 + 5 G > T	p.?	Likely pathogenic
c.2108 G > A	p.G703D	Uncertain significance
c.2522_2528del	p.I841Sfs*119	Pathogenic

Patient E is the third oldest in the CD/CRD cohort and has a relatively well preserved visual function (Fig. 4). He is homozygous for a missense variant (c.296 A > G/p.E99G). One missense variant (c.1448 A > G/p.E483G) was found *in trans* configuration with a frameshift deletion (c.2522_2528del/p.I841Sfs*119), while another (c.2108 G > A/p.G703D) is suspected, but not proven, to be *in trans* with a canonical splice site variant (c.639 + 1delG). The respective patients (families B and F) show a more severe phenotype when compared with patient E. However, the number of patients and variants in our cohort is too small to conclude that missense variants in CDHR1 have a less severe effect on protein function than frameshift and canonical splice site variants.

### Non-canonical splice site variants

Three patients in our cohort showed comparably mild phenotypes (D, G and H). In fact, the affected patient from family G, who is compound heterozygous for the non-canonical splice site variant c.783 G > A, and the in frame deletion variant c.1311_1316del/p.L437_T438del, was diagnosed with RP as an incidental finding quite late in his life. His phenotype shows a rather slow progression (see Fig. 4) which suggests that either one or both *CDHR1* variants have only a moderate effect on protein function. Since patient H is homozygous for the non-canonical splice site variant c.783 G > A, and also presented with a mild phenotype, we conclude that the non-canonical splice site variant c.783 G > A provokes only a subtle change on protein function. Performing splicing assays for the c.783 G > A variant, we could observe that transfection with the mutant allele resulted in a single transcript lacking exon 8. Skipping of exon 8 (144 bp) does not change the reading frame of the transcript, but the deletion of 48 amino acid residues would interfere with the formation of the second and third cadherin repeat. It seems likely that such a shortened protein would not be fully functional. We therefore consider the c.783 G > A variant to be pathogenic, albeit provoking only a mild phenotype due to supposed residual protein function. Of note, we also observed significant levels of exon 8 skipping (see Fig. 2A and B) with the wild type c.783 G minigene construct. Since the canonical splice donor site of exon 8 is predicted to be slightly weaker than that of exon 9 (see Supplementary Fig. S1) this might render exon 8 prone to exon skipping in this *ex vivo* assay. However, there are no human transcripts or spliced ESTs lacking exon 9 in public databases. We therefore specifically checked for alternative splicing of exons 7–9 in the human retina using RNA-seq datasets from three human healthy donors including one being heterozygous for the rare c.783 G > A variant (MAF = 0.34% in ExAC). Quantitative visualization of mRNA sequencing reads aligned to gene annotations was performed using IGV-2.3.40 (Broad Institute). The two samples homozygous for the c.783 G allele showed virtually no reads directly linking exon 7 and exon 9 (see Supplementary Fig. S2). In contrast, the retina sample heterozygous for the c.783 G > A variant showed a substantial number of reads linking exon 7 to exon 9 (see Supplementary Fig. S2). Subsequent cDNA analysis confirmed skipping of exon 8 in this sample (see Supplementary Fig. S3). Moreover we observed a markedly reduced expression of the mutant *CDHR1* allele as demonstrated by pyrosequencing-based allelic quantification (see Supplementary Fig. S4) although skipping of exon 8 does not introduce a pre-mature termination codon (but an in frame deletion) that may trigger NMD. We therefore conclude that the c.783 A variant represents a hypomorphic allele associated with reduced levels of *CDHR1* transcripts (and eventually CDHR1 protein) that may be un-masked as being deleterious if occurring in homozygous state or in heterozygous state with another pathogenic allele.

Similar to patients G and H, patient D, despite being the second oldest in the CD/CRD cohort, has a well preserved visual function suggestive of a slow disease progression (see Fig. 4). She carries the nonsense variant c.928 C > T/p.Q310* and the non-canonical splice site variant c.2040 + 5 G > T. We hypothesized that residual protein function is conferred by the non-canonical splice site variant c.2040 + 5 G > T. To confirm this assumption, we performed splicing assays for the c.2040 + 5 G > T variant which revealed two aberrantly spliced transcripts as well as a minor portion of correctly spliced transcript. Whereas the minor aberrant transcript lacks exon 16, the major one lacks both exon 15 and 16. In the latter case the predicted result is an out-of-frame deletion. It is likely that the generation of a premature stop codon will lead to nonsense-mediated mRNA decay (NMD) in this transcript^30^. The skipping of exon 16 alone does not change the reading-frame. If translated, this transcript would lead to a protein that is 86 amino acids shorter than the wildtype protein and lacks the last of six cadherin repeats. Whether this protein would be fully functional is unclear. The mechanism through which the c.2040 + 5 G > T variant leads to skipping of exon 16 in one transcript and to skipping of both exon 15 and 16 in another transcript is not known, but the skipping of either one or two exons is not restricted to *ex vivo* splicing assays. Similar complex outcomes of splicing defects have also been repeatedly reported in cDNA analyses from RNA derived from patients, for instance for *USH2A* and *ABCA4* ^31, 32^. The molecular mechanism is not quite understood but most probably can be attributed to non-sequential intron removal that is influenced by disrupted splice sites^33, 34^. One also has to consider that our minigene amplicon for the analysis of the c.2040 + 5 G > T variant due to size limitations comprises only exon 15, intron 16 and exon 16 plus adjacent intronic sequences which is of course taken out of the genomic context and thus artificial. It has been demonstrated before that the design of a minigene construct can influence the splicing outcome. Two recent studies that analyzed the very same canonical splice site mutation in the *CYP11B1* gene but utilized different minigene amplicons (one containing only two exons, the other four exons) observed very different missplicing outcomes: whereas the shorter minigene amplicon revealed intron retention for the mutant allele, the longer minigene amplicon led to either skipping of one exon or two exons^35, 36^. To add even more complexity, we observed a small amount of correctly spliced transcript for the c.2040 + 5 G > T variant which might lead to residual protein function. In addition, it is unclear whether the protein derived from the transcript with the exon 16 skip would be functional. However, this does not necessarily exclude the pathogenicity of the c.2040 + 5 G > T variant. A recent comprehensive study, in which minigene assays were performed for 17 putative splice variants in six different genes, showed an almost 100% concordance with patient RNA analyses^37^. If we therefore assume that the distribution of aberrant and correct transcripts for the c.2040 + 5 G > T variant in HEK 293 T cells reflects the situation in photoreceptors, the relatively low abundance of both the correctly spliced product and the transcript lacking exon 16 might not be sufficient to counterbalance the effect of the transcript that lacks exon 15 and 16.

In summary, we report eleven potentially pathogenic variants in *CDHR1* which were all novel at the time of their identification. Our study provides a detailed description of the clinical phenotype in 13 patients and further highlights the broad variability of *CDHR1* mutations with respect to disease onset and primary insult.

## Materials and Methods

### Patient enrollment and retrieval of blood samples

Thirteen patients from nine families were recruited and clinically examined at the Eye Hospital, Ludwig Maximilians University Munich, Germany (family A, see Fig. 1); at the Scheie Eye Institute, University of Pennsylvania, USA (family B, see Fig. 1); and at the Centre for Ophthalmology, University of Tuebingen, Germany (families C-I, see Fig. 1). Genomic DNA of patients was extracted from peripheral blood using standard protocols. Samples from all patients and family members were recruited in accordance with the principles of the Declaration of Helsinki and were obtained with written informed consent accompanying the patients’ samples. The study was approved by the institutional review board of the Ethics Committee of the University Hospital of Tuebingen.

### Clinical evaluation

Patients underwent ophthalmic examination including detailed medical history, best corrected visual acuity (BCVA) testing, kinetic and/or static perimetry, slit lamp examination, fundus examination with photography, and electroretinography (ERG). Color vision was examined by the Farnsworth D-15 test or the Arden color contrast test. In addition, several patients were tested using optical coherence tomography (OCT), fundus autofluorescence (FAF) or dark-adapted fullfield stimulus threshold (FST) test.

### Homozygosity mapping (only performed in family A)

Affymetrix CytoScan HD Arrays (Affymetrix, Santa Clara, CA, USA) were used for genotyping and regions of homozygosity were calculated with the online tool Homozygosity Mapper (http://www.homozygositymapper.org/).

### Panel-based next generation sequencing

Families B-I were screened using a custom capture panel targeting 105 retinal disease (RD) genes. Details of panel design, library preparation, sequencing and variant calling have already been published^26^.

### Sanger sequencing

Mutation screening of the *CDHR1* gene (RefSeq: NM_033100) in family A and validation of NGS called variants in the other families, respectively, was performed by bidirectional Sanger sequencing. If possible, variants were also tested for co-segregation within kinships. Gene specific PCR primers were designed and used to amplify individual exons and flanking intron sequences applying standard PCR amplification protocols. Primer sequences are available upon request. PCR fragments were purified by ExoSAP-IT treatment (USB, Cleveland, OH), sequenced using Big Dye Termination chemistry (Applied Biosystems [ABI], Weiterstadt, Germany) and products separated on a DNA capillary sequencer (ABI 3100 genetic analyzer; ABI, Weiterstadt, Germany).

### *In silico* analysis

The potential pathogenicity of the missense changes identified in this study was assessed using four online prediction software tools, namely SIFT [http://sift.bii.a-star.edu.sg/], PolyPhen-2 [http://genetics.bwh.harvard.edu/pph2/], Mutation Taster [www.mutationtaster.org/] and Provean [http://provean.jcvi.org/]. In addition, the Exome Aggregation Consortium (ExAC) Browser [http://exac.broadinstitute.org/] was queried for the presence and minor allele frequencies (MAF) of identified variants. The following five splice site prediction programs were used to predict the effect of variants on transcript splicing: Splice Site Finder (http://www.interactive-biosoftware.com); GeneSplicer (http://www.cbcb.umd.edu/software/GeneSplicer); Splice Site Prediction by Neural Network (http://www.fruitfly.org/seq_tools/splice.html); MaxEntScan (http://genes.mit.edu/burgelab/maxent/Xmaxentscan_scoreseq.html); and Human Splicing Finder (http://www.umd.be/HSF/). All *in silico* analyses with the exception of Provean were performed by the integrated software Alamut v.2.7.1 (http://www.interactive-biosoftware.com) using default settings in all bioinformatic tools.

### Minigene assays

A 4.0-kb fragment comprising *CDHR1* exon 7 with 396 bp of upstream sequence, intron 7, exon 8, intron 8, and exon 9 with 773 bp of downstream sequence was amplified using a proofreading polymerase and genomic DNA from the affected proband of family J (see Fig. 1) in order to simultaneously amplify both the mutant and the non-mutant gene fragment. Likewise, a 2.2-kb fragment comprising *CDHR1* exon 15 with 329 bp of upstream sequence, intron 15, and exon 16 with 694 bp of downstream sequence was amplified from the affected proband of family G (see Fig. 1). Cloning into the exon-trapping vector pSPL3, transfection of human embryonic kidney (HEK) 293 T cells, RNA isolation and RT-PCR were performed as described previously^38^.

## Electronic supplementary material

Supplementary Information
