# Supplementary material for: CDHR1 mutations in retinal dystrophies
Source: Sci Rep. 2017 Aug 1;7:6992. doi: 10.1038/s41598-017-07117-8 (PMC5539332; doi:10.1038/s41598-017-07117-8)
Supplement: Supplementary file 1 — Supplementary Information [file 41598_2017_7117_MOESM1_ESM.pdf]

## Supplementary Information

## *CDHR1* mutations in retinal dystrophies

Authors

Katarina Stingl[1], Anja K Mayer[1], Pablo Llavona[1], Lejla Mulahasanovic[2], Günther Rudolph[3], Samuel G Jacobson[4], Eberhart Zrenner[1,5], Susanne Kohl[1], Bernd Wissinger[1], Nicole Weisschuh[1]*

## Supplementary Materials and Methods

### Tissue

All procedures conformed to the provisions of the Declaration of Helsinki for the use of human tissue in research and were approved by the Institutional Review Board of the University Clinics in Tuebingen. The previous medical and ocular histories of all donors were assessed to exclude donors with any eye disease. Three pairs of healthy adult donor eyes (age range 63–81 years) were obtained between 24 and 30 hours postmortem. To obtain fresh tissues for RNA extraction, globes were processed less than 48 hours postmortem. Dissected retinal tissue was immediately submerged in RNA Stabilisation Reagent (RNA later; Qiagen).

### DNA isolation, RNA isolation, cDNA synthesis and reverse transcription PCR

DNA was isolated from the sclera using the PeqGold Tissue DNA MiniKit (PeqLab), following the manufacturer's instructions. Total RNA was extracted from retina using the RNeasy Mini Kit (Qiagen), following the manufacturer's instructions. RNA was quantified using a NanoDrop ND-1000 spectrophotometer (NanoDrop Technologies) and the integrity was evaluated using an RNA 6000 Nano chip on a Bioanalyzer (Agilent Technologies). The RNA of all three samples was sufficiently intact with an average RNA Integrity Number (RIN) of 6.83 ± 0.17. cDNA was synthesized using the Transcriptor High Fidelity cDNA Synthesis Kit (Roche Applied Science) with 500 ng input RNA and 600 pmol/µl of Random Hexamer primers. The cDNA was PCR amplified with *CDHR1* primers located in exon 7 (forward primer: GGCCACTCTGGACTACGAGA) and exon 9 (reverse primer: GCGACCACCTTCAGTACCTC) using standard PCR protocols.

### Library preparation and sequencing

For RNA-seq analysis, RNA libraries were constructed using a TruSeq small RNA sample preparation kit (Illumina), following the manufacturer's protocol. The sequencing was carried out in collaboration

with the core facility c.ATG at the Institute of Medical Genetics and Applied Genomics in Tuebingen. Sequencing was performed on the HiSeq1000 platform (Illumina).

**Computational analysis**

RNA-seq data from three healthy human retinas was aligned using hg19 as reference genome. Broad Institute Integrated Genomics Viewer (IGV-2.3.40, Cambridge, MA, USA) was utilized to visualize aligned reads. Sashimi plots of *CDHR1* were generated to distinguish differentially spliced exons in each of the three healthy human retina transcriptomes.

**Pyrosequencing assays**

PyroMark Assay Design (Qiagen) was used to design primer sequences. Pyrosequencing was performed on the PyroMark Q96 ID (Qiagen) followed by data analysis with PyroMark™ ID (Qiagen). To evaluate transcript relative allelic abundance of exon 8 at nucleotide position c.783 in retina derived cDNA, a forward primer (5'biotin-GTCTTCGTGGGCACACCCTACTAT) was designed that binds within exon 8. The reverse primer (TTTGCCCCGGTCTCCATC) and the sequencing primer (TTCAGTACCTCCGAGC) bind within exon 9. A second pyrosequencing assay had to be designed to evaluate allele ratios on genomic DNA level since nucleotide position c.783 is at an exon-intron junction. To this end, a pyrosequencing assay was designed that interrogates SNP rs4933980 in exon 17 (forward primer: TCTCATCCTGACCCCTCTGTCT; reverse primer: 5'biotin-AGGGTGTCACCATCATGAAATAGC; sequencing primer: GCTTCTTTTGCTTGGC). This assay was performed on DNA extracted from human sclera. Three replicates were performed for both assays.

## Supplementary Figures

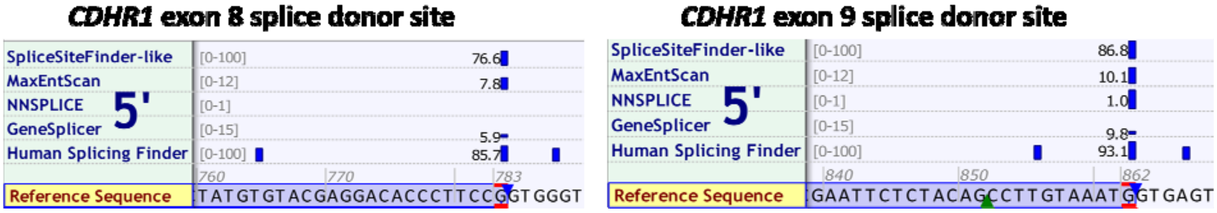


**Supplementary Figure S1:** *In silico* prediction of the strength of *CDHR1* exon 8 and exon 9 splice donor sites. The scores of the five algorithms used to assess the effect on splicing are depicted as blue bars. The canonical splice donor site of exon 9 is predicted to be slightly stronger than that of exon 8.

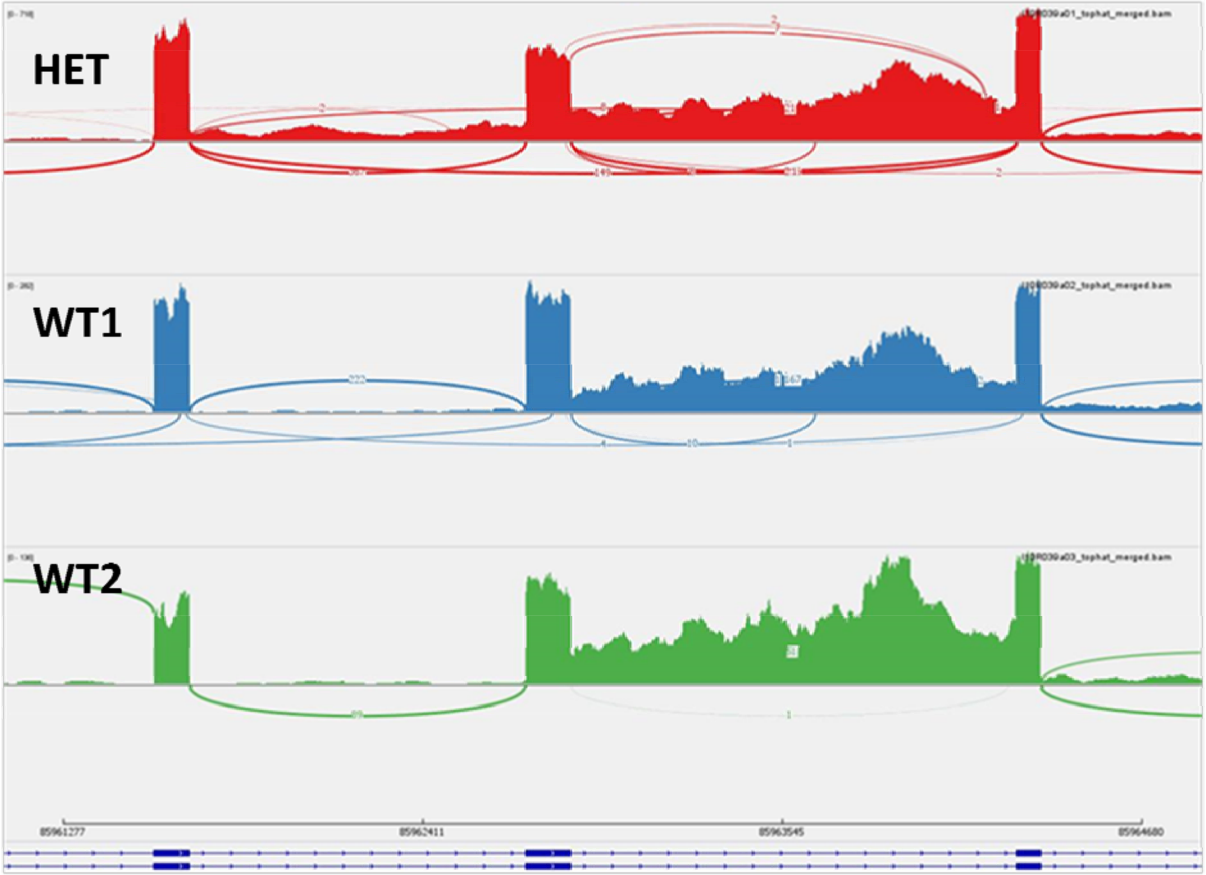


**Supplementary Figure S2:** Sashimi plots of exon 8 and its flanking exons in *CDHR1*. Sashimi plots were obtained from RNA-Seq data of one retina sample being heterozygous for the c.783G>A variant (HET) and two samples being homozygous for the wild type G allele at position c.783 (WT1 and WT2). The arcs indicate splice junction reads, with the thickness of the arc correlating with the number of junction reads spanning the two exons being connected by the arc. A significant number of reads (n=149) between exon 7 and 9, indicating skipping of exon 8, are only seen in sample HET.

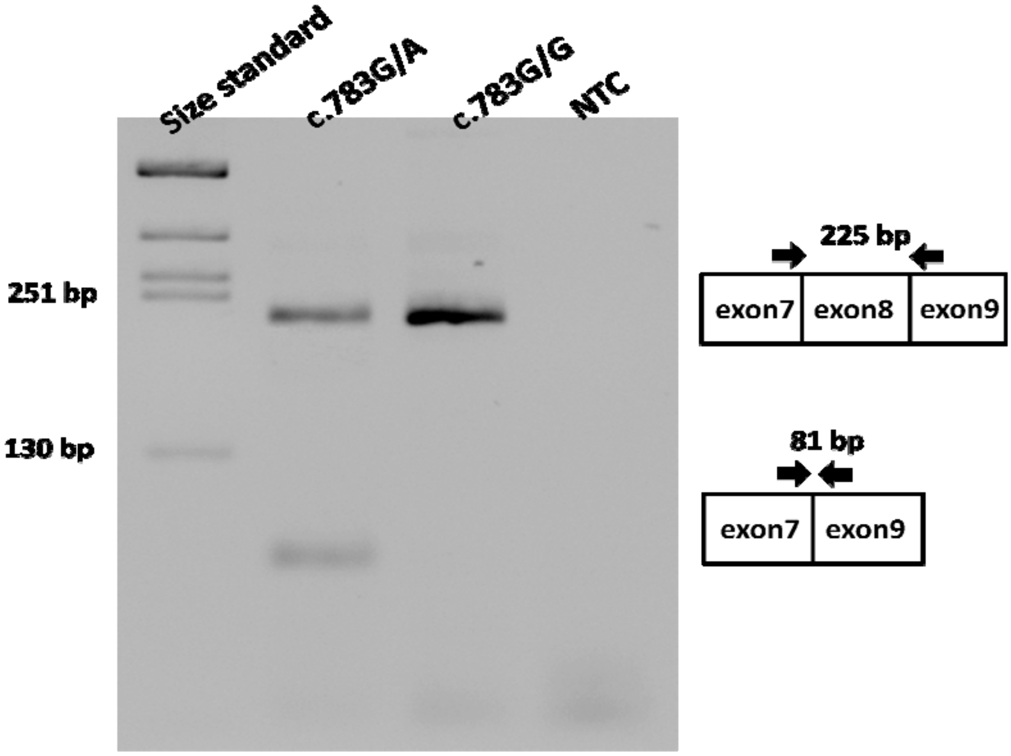


**Supplementary Figure S3:** cDNA analysis of human retina. RT-PCR using *CDHR1* specific primers revealed two transcripts for a human eye donor being heterozygous for the c.783G>A variant. Another eye donor, who was shown to be homozygous for the wild-type G allele, showed only one transcript. The bigger transcript includes exon 8 whereas the smaller transcript skipped exon 8. Schemes of the amplified products are presented on the right of the agarose gel. Arrows indicate the binding sites for the primers used in the RT-PCR. NTC, non template control.

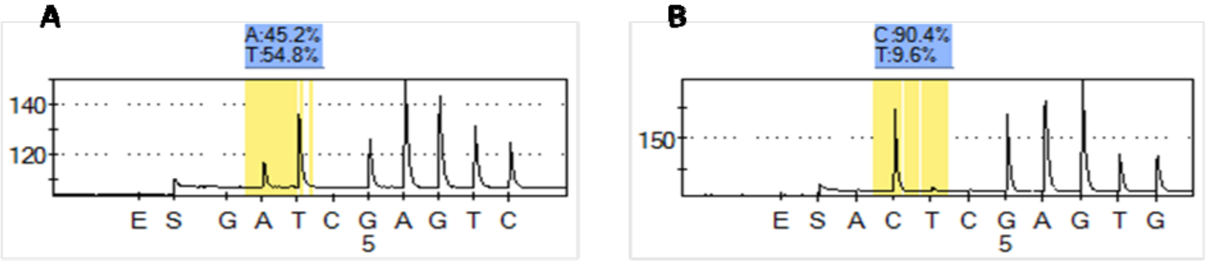


**Supplementary Figure S4:** Pyrosequencing assays of genomic DNA and cDNA of a sample being heterozygous for the c.783G>A variant. (a) Representative output trace (pyrogram) from genomic DNA showing nearly equal allele ratios for the interrogating SNP rs4933980. The SNP investigated in the assay is highlighted in yellow. The height of each peak indicates the amount of each nucleotide incorporated. Subsequent computing considers the sequence of neighboring nucleotides and calculates the percentage of each allele present which is indicated in the blue box. (b) Representative pyrogram from cDNA showing strong allelic imbalance with a ratio of 9:1 in favor of the transcript derived from the wild type allele.
